# Thinking outside the box means thinking outside the search engine

**DOI:** 10.3758/s13421-025-01732-x

**Published:** 2025-05-28

**Authors:** Daniel M. Oppenheimer, Mark T. Patterson

**Affiliations:** Department of Social and Decision Sciences, Carnegie Mellon University, 4851 Frew St, Pittsburgh, PA 15213 USA

**Keywords:** Creativity, Alternative uses task, Internet access, Digital tools, Nominal groups

## Abstract

This study investigates the impact of internet access on creativity and identifies potential hidden costs of internet use for groups. Using the alternative uses task, we randomized participants (*N* = 244) into separate conditions to generate ideas for nonstandard uses for one of two common objects—a shield or an umbrella—either with or without internet access. Nominal group analysis reveals that while individual creativity may be enhanced by internet access, groups articulate fewer novel solutions when provided internet access, suggesting that internet access may constrain collective creative fluency. We also ran a reanalysis of previous data sets on creativity and internet use and found robust converging evidence across different paradigms, coders, and contexts. We further explore robustness by examining alternative operationalizations of fluency: quality of responses, as measured by coders’ evaluations of effectiveness, novelty, and subjective evaluations of creativity. While overall trends suggest an advantage for subjects who do not have internet access, this patterning depends to some degree on variation among coders. Implications for the way digital tools influence creative processes are discussed.

When humans use tools, it changes the way they think. Different technologies have different cognitive affordances, which guide people toward different cognitive schemas/representations, different cognitive processing strategies, and different social dynamics. Thus, for better understanding how technology will affect society, we need to understand not just how technology functions, and not just how humans function, but how the extended organism of humans interacting with technology will function (cf. Hamilton & Benjamin, 2019).

In light of this, researchers have explored how digital technology is influencing various forms of human cognition, including memory (Eliseev & Marsh, 2021; Sparrow & Wegner, 2011), metacognition (Fisher & Oppenheimer, 2021a, 2021b), attention (Trielli & Diakopoulos, 2019), executive control (Loh & Kanai, 2016), and most germane to the present study, higher order functions, such as creativity (Oliva & Storm, 2023; for reviews, see Frich et al., 2018, 2019).

There are multiple ways that using digital tools, such as internet search, could influence creativity. Online resources could provide users with ideas that might not otherwise come to mind. Any idea that Google displays that a person would not have thought of independently adds to the total number of ideas generated. Moreover, such an idea might serve as a prompt, priming the person to generate other ideas that otherwise might not have come to mind.

That said, exposure to external ideas can actually constrain, rather than enhance, creativity. Studies have shown that when participants are asked to generate ideas, their performance declines if they are provided with examples (Smith et al., 1993). Working in groups can inhibit creativity because receiving ideas from others reduces the range of ideas that participants generate (e.g., Kohn & Smith, 2011). Indeed, studies of brainstorming often show “production blocking,” wherein the number of ideas generated by people brainstorming in actual groups is lower than the number of ideas generated by nominal groups (i.e., aggregating the ideas of people brainstorming by themselves; Diehl & Strobe, 1987; Nijstad et al., 2003; Taylor et al., 1958). Indeed, in the human–computer interaction literature, scholars have developed tools to overcome “fixation,” wherein people get anchored on a particular mental set and are unable to explore the full space of creative possibilities (e.g., Kerne et al., 2014).

A recent study by Oliva and Storm (2023) explored how access to the internet could affect creativity. Participants performed the alternative uses task (Guilford, 1957), in which they generated nonstandard uses for everyday items. Some participants had access to the internet while others did not. Oliva and Storm found no differences between conditions across a number of dependent variables: fluency (number of ideas generated), creativity, novelty, flexibility, or uniqueness, although they did find that internet users’ answers were more “effective.”

While this study represents an important step in understanding how using the internet affects creativity, it leaves an important question unanswered. Specifically, the study did not explore collective creativity. In many studies of brainstorming, the unit of analysis is not the performance of an individual, but the performance of groups and nominal groups (e.g., Diehl & Strobe, 1987; Nijstad et al., 2003; Taylor et al., 1958). Indeed, in a seminal review of the literature on digital tools and creativity, over a third of the nearly 1,000 papers explored focused on collaborative creativity (Frich et al., 2018). Using the internet may not hurt creativity at the level of the individual, but to the extent that people using the internet are all primed by similar examples, they may come up with similar ideas and consequently have less diversity of ideas as a collective than people who are not using the internet. The purpose of the present investigation is to explore whether that is indeed the case. To do this, we ran a novel study on how internet use affects creativity for both individuals as compared with nominal groups, and we reanalyze the data from Oliva and Storm (2023) studies to extend the results to nominal groups.

Our study will also explore how creativity is impacted as a function of how easy it is to find relevant suggestions on the internet. For example, the most famous version of the alternative uses task asks participants to generate uses for a brick. Consequently, an internet search for creative uses of bricks reveals databases of participant answers from dozens of studies—far more than a person could conceivably generate on their own, given the time constraints of a lab study. Before May 2024, searching for creative uses of knee braces yielded exactly one suggestion: supporting injured knees. The Google AI Overview now incorporates LLM results (drawn from Gemini), and requests for alternative uses for nontypical items are now sometimes met with plausible use cases.^1^ Importantly, data collection for our study occurred before the AI supplemented Google search. Oliva and Storm (2023) did not describe why they chose the particular prompts that they used (brick, nail, paperclip, safety pin), but all four are objects with websites devoted to suggesting creative uses show up early in a Google search. Here, we look at how results are affected by the number of suggestions that Google search provides.

Finally, the original Oliva and Storm (2023) article limited participants to generating a maximum of five creative uses for an object. This is because the primary goal of the Oliva and Storm was not to evaluate the number of items that participants could generate (i.e., “creative fluency”; although they did measure creative fluency in their paper) but rather to measure potential differences in the type and creativity of uses generated. Nonetheless, for the purpose of understanding creative fluency, this five-item cap could have led to ceiling effects or otherwise distorted the distribution of creative ideas that might have been generated had there been no externally imposed limit. As such, in the present study, we do not artificially limit the number of items to be generated, aside from the constraints of the time that participants are given to work on the task.

## Study 1

### Method

#### Participants

A total of 256 participants were recruited at (redacted for blind review) in exchange for course credit. The sample was 60% female, 37% male, with the remainder either expressing other gender identities or not disclosing gender identity. The participants were 56.5% Asian, 22.5% White, 7.5% mixed race, 7% Hispanic, and 4% Black, with the remainder either not disclosing or expressing other racial identities. Participants were university undergraduates between the ages of 18 and 22 years. The sample size was determined using an “as many as we could get before the subject pool closed” strategy. The resulting sample size of 256 provided a 98% chance of detecting a moderate effect size (*d* = 0.5) and an 80% of detecting a small/moderate effect size (*d* = 0.3). We did not look at or analyze the data until the study pool had closed and all participant data had been run.

### Stimuli and procedure

Participants completed the study as part of a battery of unrelated studies on note-taking and ignoring distractions. Participants performed a variant of the classic “alternative uses task” paradigm (Guilford, 1957). Participants were asked to come up with as many uses for a common object as they could within 3 min. Answers were typed into a text box, and a timer tracking the time remaining was prominently displayed on the screen. However, neither the timing and keystroke records of when they entered uses (e.g., when they typed their first entry, their last entry, and whether there were any clusters of entries) nor click data was recorded. At the end of 3 min, participants were automatically taken to the next study.

Participants were randomly assigned to one of two conditions: In the Google condition, participants were instructed to “use Google and anything linked in its search results” to come up with ideas. Participants in the no-Google condition were told to “stay on the survey site” and not go to other websites. To ensure that participants were following the instructions, the studies were run in person, with a research assistant discreetly observing participants’ internet use. However, neither the internet search terms used nor the webpages viewed were recorded.

Participants were randomly assigned to generate as many uses as they could for either an umbrella or a shield. These objects were selected based on two criteria. First, neither had been used in previous alternative uses task studies with full datasets available on the internet (i.e., participants could not look up uses generated in previous studies). Second, these objects varied in terms of what a Google search for “uses for a {shield/umbrella}” revealed. At the time of data collection, searching Google for uses of a shield yielded two suggested uses: to block attacks, or to hit somebody with during combat. Searching Google for uses of an umbrella, the first hit is “10 different ways to use your umbrella (2024),” which includes a walking stick, decoration, self-defense, and caring for plants (in addition to protecting oneself from rain). This allows us to explore how creativity changes as a function of the number of uses that Google is likely to suggest.

### Results

#### Subject filtering

After data collection, 12 subjects were removed due to technical malfunction of the survey or question misunderstanding. After subject removal, there were 121 subjects in the shield condition (58 in the Google, 63 in the non-Google group), and 123 subjects in the umbrella condition (68 in the Google, 55 in the non-Google group).

#### Category coding

A research assistant who was blind to the experimental hypothesis and whether a participant had access to the internet coded the responses into categories of distinct uses (e.g., “use as a weapon,” “to hit somebody,” and “attack with it” would all be given the same category code, reflecting that those three uses are conceptually the same). The coder was given full discretion over what counted as a category, so as to avoid experimenter expectancy effects biasing the classification scheme.^2^ Classification revealed 78 distinct categories for shields and 92 distinct categories for umbrellas.

#### Individual use generation

For generating uses of a shield, there were no significant differences between the Google group (*M* = 5.88, *SD* = 3.92) and the non-Google group (*M* = 6.46, *SD* = 3.87; *t* = 0.82; *p* = 0.41), conceptually replicating the findings of Oliva and Storm (2023).

However, for uses of an umbrella, subjects in the Google group generated significantly more uses (*M* = 9.72, *SD* = 3.51) compared with the non-Google group (*M* = 7.76, *SD* = 3.19; *t* = 3.20; *p* < 0.002). In other words, when Google provided few suggestions, it was not helpful, but when Google provided many suggestions, it was helpful. Importantly, means in all four conditions were higher than the cap of five generated uses that Oliva and Storm (2023) imposed on their sample, suggesting that their null results for fluency could be partly due to a ceiling effect cutting off the full range of the distribution.

#### Nominal groups use generation

Separately, for the Google and non-Google groups, we created nominal groups by drawing 10,000 samples of groups of size *k* (without replacing individual participants within a sample) for groups of varying size (*k* ranging from 1 to 20). We then calculated the average number of distinct uses generated across all group members for each nominal group.

**Fig. 1:**
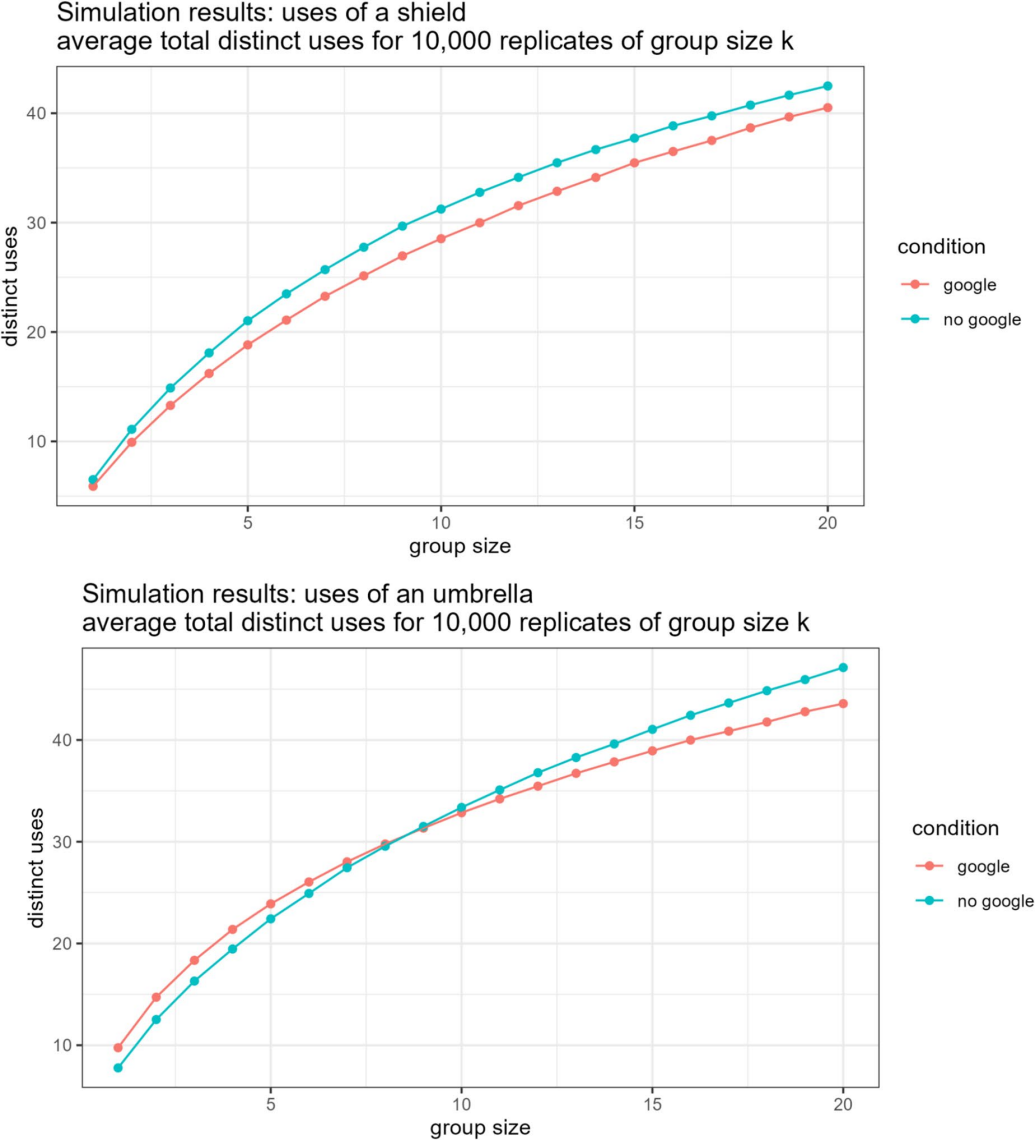
Nominal group use generation results

Figure 1 shows the results from the nominal group construction. For uses of a shield, nominal groups of any size generated more distinct uses when composed of non-Googling rather than Googling subjects. For uses of an umbrella, nominal groups of small size (*k* < 9) composed of Googling subjects outperform non-Googling nominal groups, but a greater number of distinct uses were generated by non-Googling nominal groups with at least nine subjects. In other words, when Google does not yield useful results, nominal groups do better without Google. When Google does yield useful results, small nominal groups do better with Google, but as group size increases, that advantage shrinks and eventually reverses.

#### Group composition of uses by response frequency

**Fig. 2:**
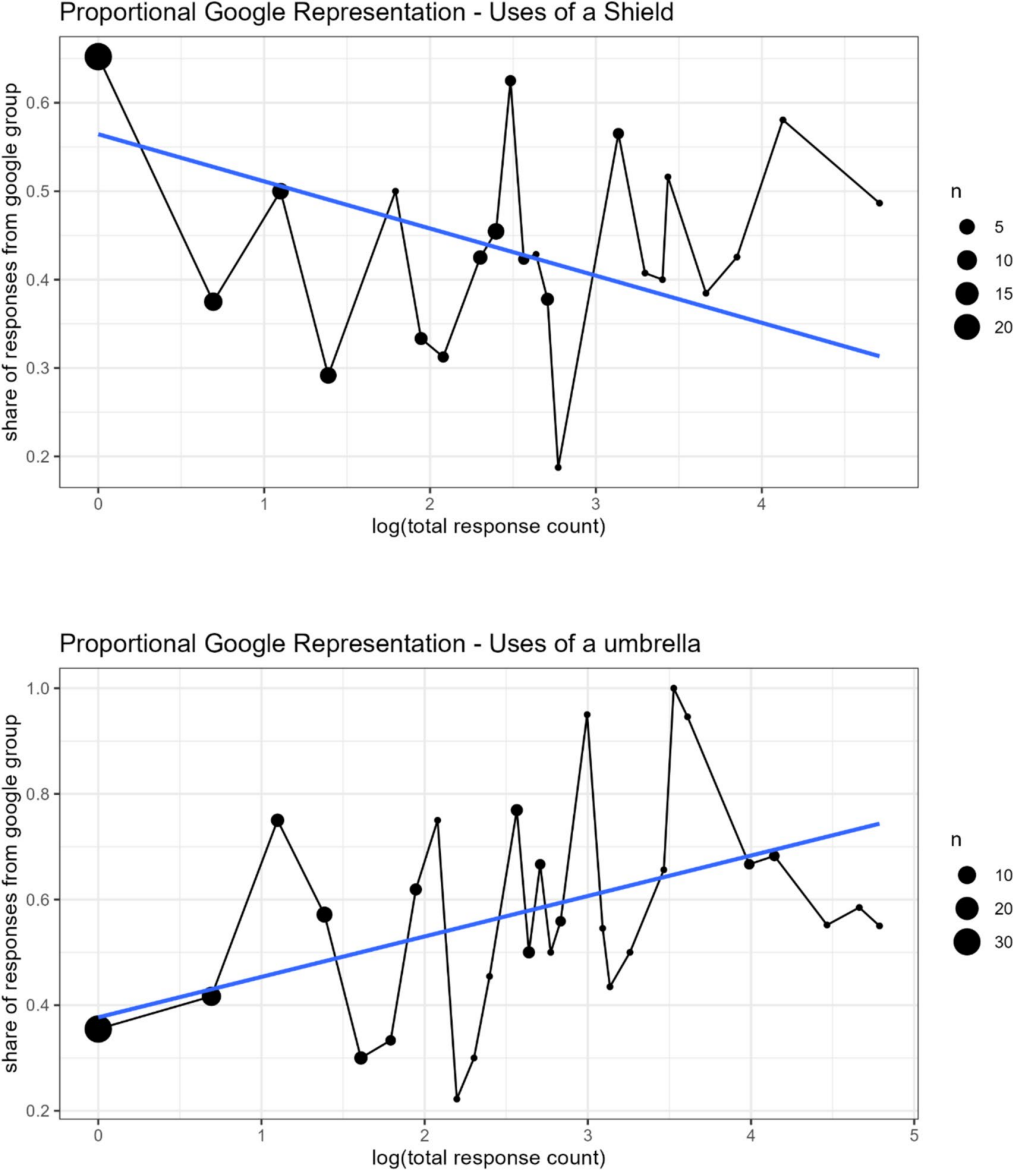
Proportional group representation of uses

In addition to counting the total number of distinct uses generated, we analyze compositional differences (Google vs. non-Google) when data are grouped by the total frequency of uses. As an example, subjects generating uses of an umbrella produced 31 “singleton” responses, meaning that only one subject thought of each use. Of the 31 singleton responses, 20 (64% of the total) were generated by subjects in the non-Google group, and 11 (36%) were generated by subjects in the Google group. Figure 2 presents a summary of the compositional representation when uses are grouped by the log total response count; the linear model (weighted by total responses) is added to each plot. In other words, if there were 10 doubleton responses, that would mean that 20 participants provided a doubleton response (2 participants × 10 responses); we explore what percentage of those 20 came from the Google versus non-Google conditions.

Regression analysis^3^ revealed a systematic positive relationship between these features in the umbrella condition (β = 0.08, *p* < 0.001). The pattern indicates that subjects with access to Google, when generating uses of an umbrella, overreport commonly identified uses relative to the generation of novel, distinctive uses. Meanwhile, regression shows a significant negative association between log response count and Google proportion in the shield condition (β =  − 0.05, *p* = 0.02). Unexpectedly, when generating uses of a shield, subjects with access to Google tend to report less commonly identified uses. We will return to this finding in the discussion. These results are robust to variants in the regression model (e.g., log data vs. raw data).

#### Ordering on “first three” responses

As a final measure of commonality in use generation, we present a simple measure of patterning in the order of uses listed. Table 1 presents frequency counts of the most common “first three” responses listed. (In cases where respondents generate fewer than three responses in total, the set of responses generated is still treated as a triple). Frequency counts of such common response patterns in the shield condition suggest no differences when subjects have access to Google, but in the umbrella condition, we observe clustering (*n* = 13 and *n* = 8 participants) in the Google groups reporting the same first three uses.

**Table 1:**
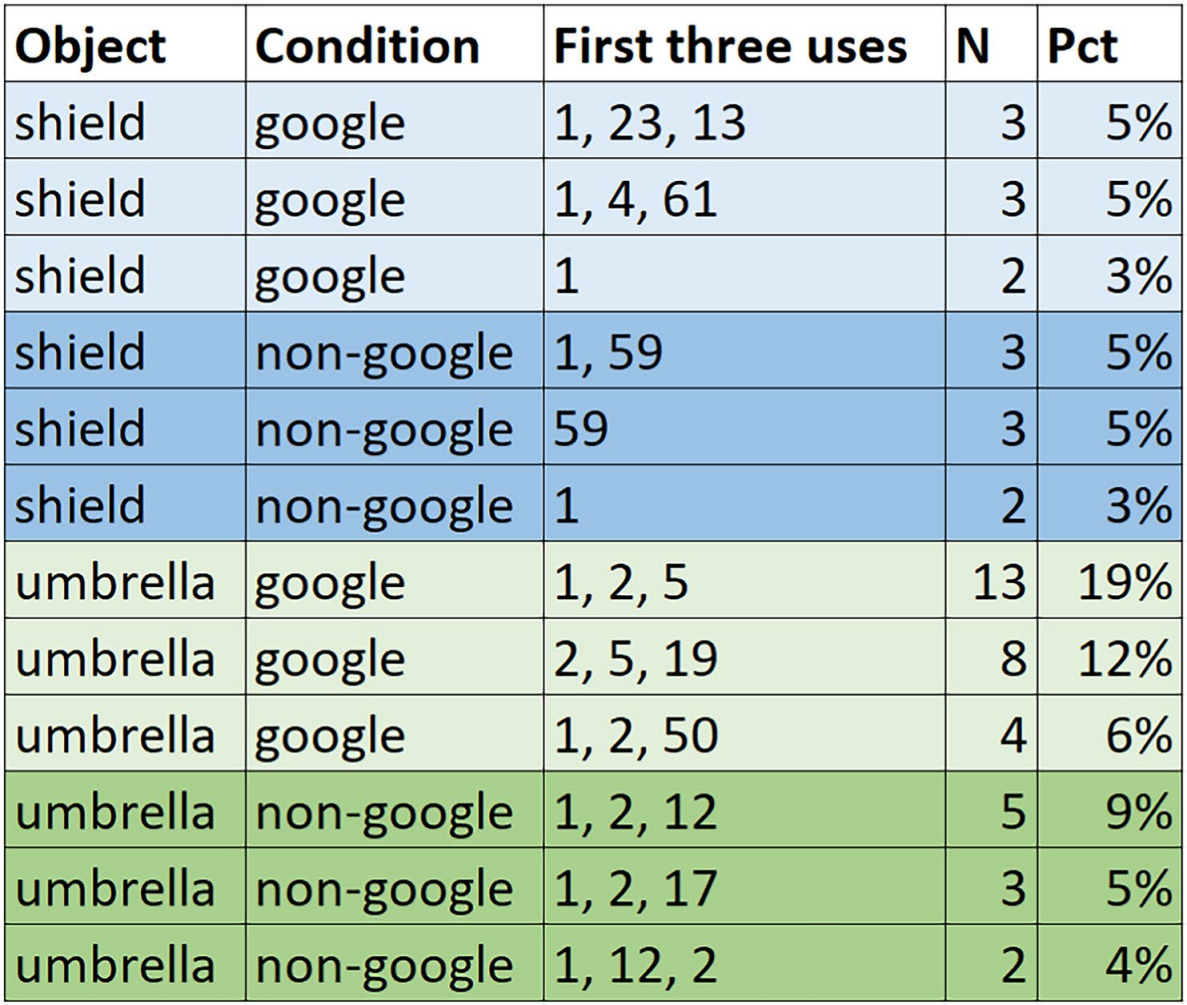
Most common “first three” uses

The numbers in the third column represent the codes for the categories. Thus, in the Google-umbrella group, 19% of participants listed Category 1, Category 2, and Category 5, in that order.

#### Operationalizations other than creative fluency

While one common way of operationalizing creativity is through creative fluency—the number of unique ideas that participants generate—it is not the only operationalization in the literature. In particular, three other approaches explored by Oliva and Storm (2023) are novelty, effectiveness, and subjective evaluations of idea creativity.

To explore how internet use affects individual and nominal group creativity using these alternate operationalizations, two coders blind to hypothesis and condition independently rated all uses that were generated in the study on these three dimensions. Coder instructions were taken from Oliva and Storm (2023)—namely, to evaluate each item on a 1 (*low*) to 5 (*high*) scale on all three constructs using the following definitions:

- **Effectiveness:** Effectiveness is defined as how well the object would serve in the purpose described by the participant and whether that use is “useful.”
- **Novelty:** Novelty is defined as how new or surprising the use is to you (the raters).
- **Creativity:** When evaluating creativity, we would like you to consider the standard definition of creativity (novelty and effectiveness) as well as the overall, amorphous creative quality of the uses being generated. In other words, please generate a single rating of creativity for each use that not only considers how novel and effective the use is but also how much it subjectively feels to you as though it has the kind of surprising or unexpected quality that is typically associated with creativity.

To assess the impact of internet access on the generation of high-quality ideas, we ran our simulations using the same nominal group structure described earlier. Specifically, we again drew 10,000 random samples of nominal groups for each group size (*k* = 1–20), for each item (shield or umbrella), ensuring that no individual participant was sampled more than once within a given group. However, rather than calculating the average number of distinct ideas generated, we instead computed the average number of high-quality ideas generated per nominal group under each of the three operationalizations (creativity, novelty, and effectiveness).

We analyze nominal group generation using two focal thresholds for high-quality ideas: (1) the count of ideas rated as 5 on the respective scale, and (2) the count of ideas rated as 4 or 5. This dual approach allows us to distinguish between the strictest possible standard of exceptional creativity and a broader definition that includes ideas that are still highly creative but may not reach the very highest rating. Our coders differed in how generous versus stingy they were in awarding scores, with one coder awarding noticeably higher scores than the other. The interrater reliability between coders was unacceptable, and the results varied in subtle ways for the different coders, so we opted to consider them separately rather than averaging their scores. Counts were calculated separately for each of the two high-quality thresholds (5 only, and 4 or 5) for each of the two coders.

## Results

**Fig. 3:**
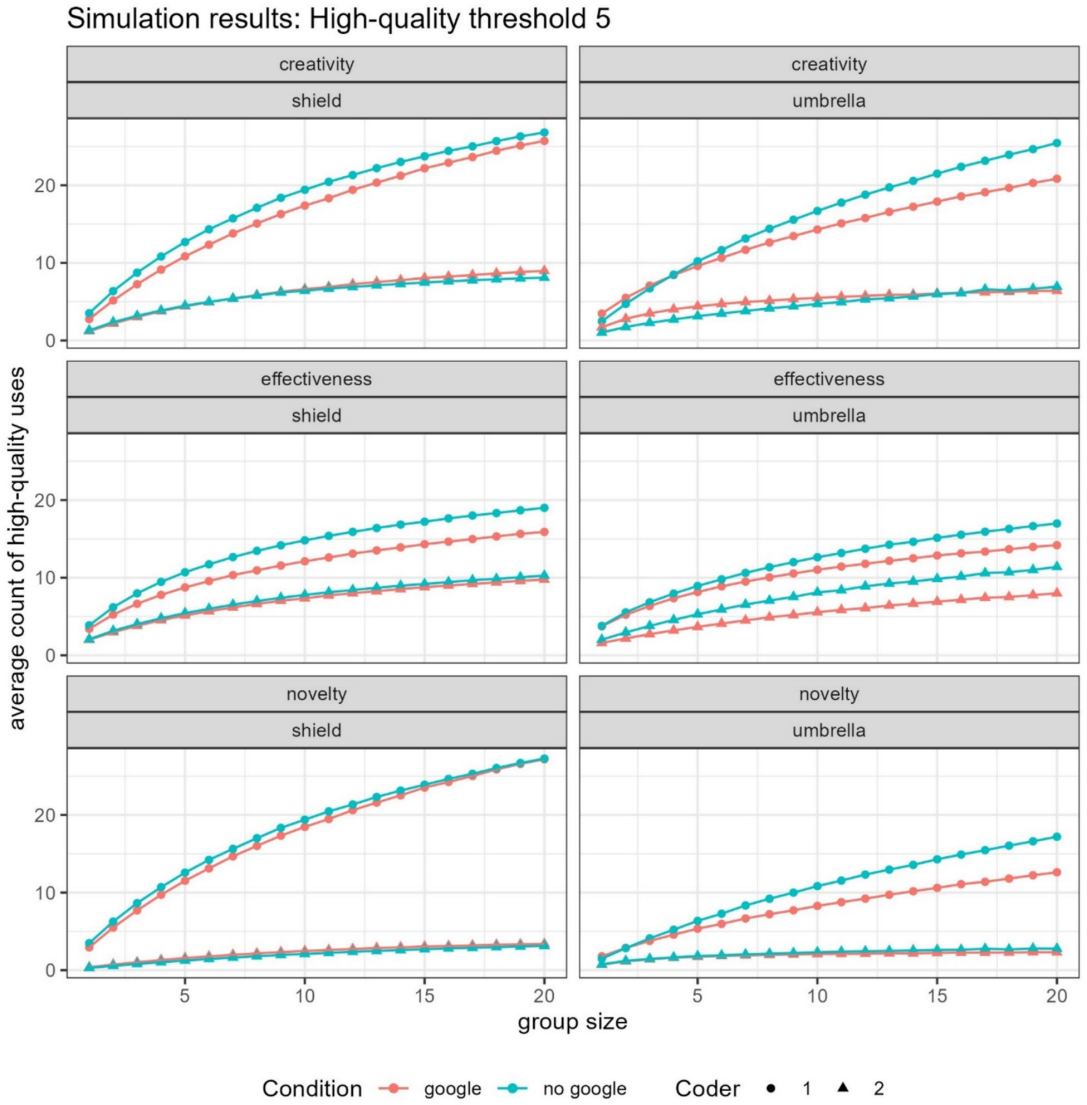

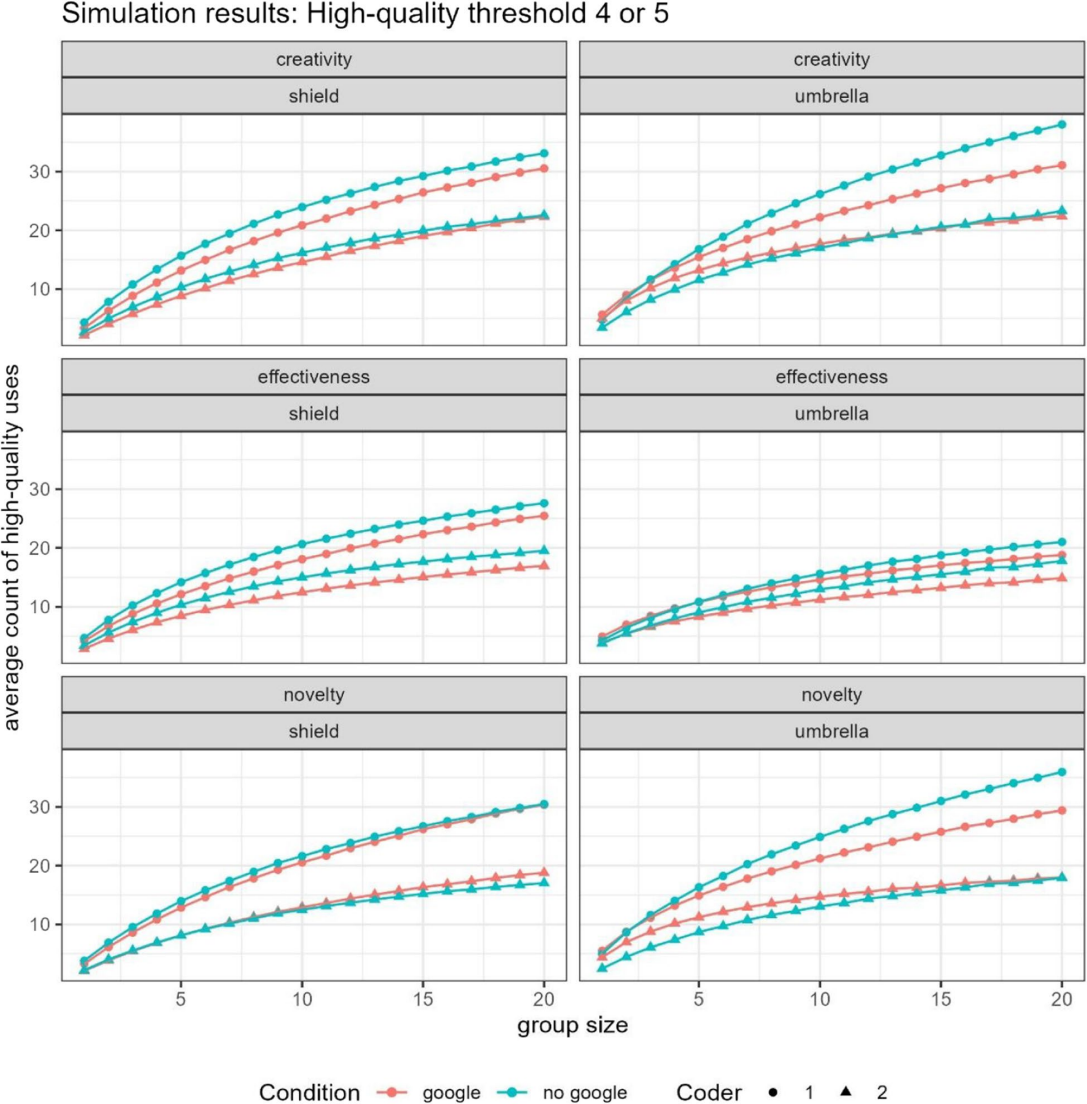
Number of observations of “high-quality” answers generated by nominal groups as a function of group size when “high quality” is coded as either a score of 5 on a 1–5 Likert scale (top panel) or as a 4 or 5 (bottom panel). The data are broken down by whether participants had access to Google (red) or not (blue), which coder was doing the evaluation (circle vs. triangle), and the object of brainstorming: shield (left) or umbrella (right). (Color figure online)

For the more lenient coder, results for the new operationalizations look qualitatively similar to the results on creative fluency. The only exception was when shields were evaluated on the “novelty” dimension, in which case there was no appreciable difference between groups that differed with respect to Google access. For all other operationalizations (novelty, effectiveness, and creativity), for both umbrellas and shields, and regardless of the threshold for “high-quality” (4 vs. 5), as nominal group size increased, lack of access to Google improved creative outcomes (see Fig. 3).

For the stricter coder, there were very few scores of 5, and this low sample size led to greater uncertainty in our estimates of the proportion. That said, for a threshold of 5, Google and non-Google groups were largely indistinguishable from each other for shields, regardless of nominal group size. For umbrellas, when creativity was operationalized as novelty or effectiveness, as group size increased, the relative advantage for groups of non-Google users over Google users increased (consistent with the other coder, and the findings from creative fluency). However, in contrast with previous findings, “subjective creativity” was largely indistinguishable regardless of whether or not groups had access to Google.

When the threshold for “high quality” was made more lenient (scores of 4 or 5 on a 5-point Likert scale), this observed advantage for Google users in “subjective creativity” for uses of umbrellas disappeared. However, the “novelty” advantage for non-Google users looking at umbrellas also disappeared. Meanwhile, for shields, with a less strict threshold, an advantage for non-Google users in “effectiveness” emerged, and an advantage for Google users in “novelty” emerged, both increasing as nominal group size increased.

In summary, the results were nuanced, depending on the threshold used for what constitutes a high-quality answer, the specific operationalization, the coder, and the object of brainstorming. However, to help simplify the interpretation of the results, we a performed a straightforward count of which type of group (with vs. without Google access) performs best—across different coders, items, and thresholds—shows that nominal groups without Google consistently beat out groups with Google. They were twice as likely to win on measures of novelty, six times as likely to win on measures of creativity, and always win or tie for measures of effectiveness (see Table 2).

**Table 2:** Summary of simulation findings: count of which type of group (with vs. without Google access) performs best—across different coders, items, and thresholds

	Google	No Google	Tie
Creativity	1	6	1
Effectiveness	0	7	1
Novelty	1	2	5

### Reanalysis of Oliva and Storm (2023)

As a robustness test on the observed patterns of results from Study 1, we reanalyze data reported by Oliva and Storm (2023). In the original study, they analyzed the data from 297 participants who completed a version of the alternative uses task for four items (brick, nail, paper clip, and safety pin) either aided by Google or not. In this version of the task, participants were asked to list up to five uses for each item. While the goals of that study were not focused on creative fluency (number of uses generated) but rather to measure potential differences in the type and creativity of uses generated found, they did report on creative fluency, finding no difference between Google users and non-Google users. That said, they only examined individual performance. Here, we examine that data under the nominal-groups paradigm to see if the trends align with those from Study 1.

### Category coding

We recruited five^4^ coders, blind to condition and hypothesis, to classify the raw responses from the original paper into distinct uses, using a coding scheme of their choice.^5^ Flexibility in the coding scheme yielded a large degree of variation in the total number of distinct uses identified. The number of uses of a nail in this data, for example, yielded 22, 66, 89, 152, and 158, distinct category codes across the coders. Natural variation in coder’s choices about what constitutes a distinct use allows us to explore robustness of the patterns and ensure that results are not an artifact of the specific coding scheme that was used.

### Individual participant-level findings

Averaging over unique combinations of participant and item, 80.7% of participants generated five uses—the maximum number allowed in the study paradigm. These high numbers, taken in conjunction with the findings from Study 1, suggest that there may have been a ceiling effect in the original Oliva and Storm (2023) study, possibly explaining the lack of variation in fluency (i.e., generated uses) that was observed between conditions.

### Nominal group findings

**Fig. 4:**
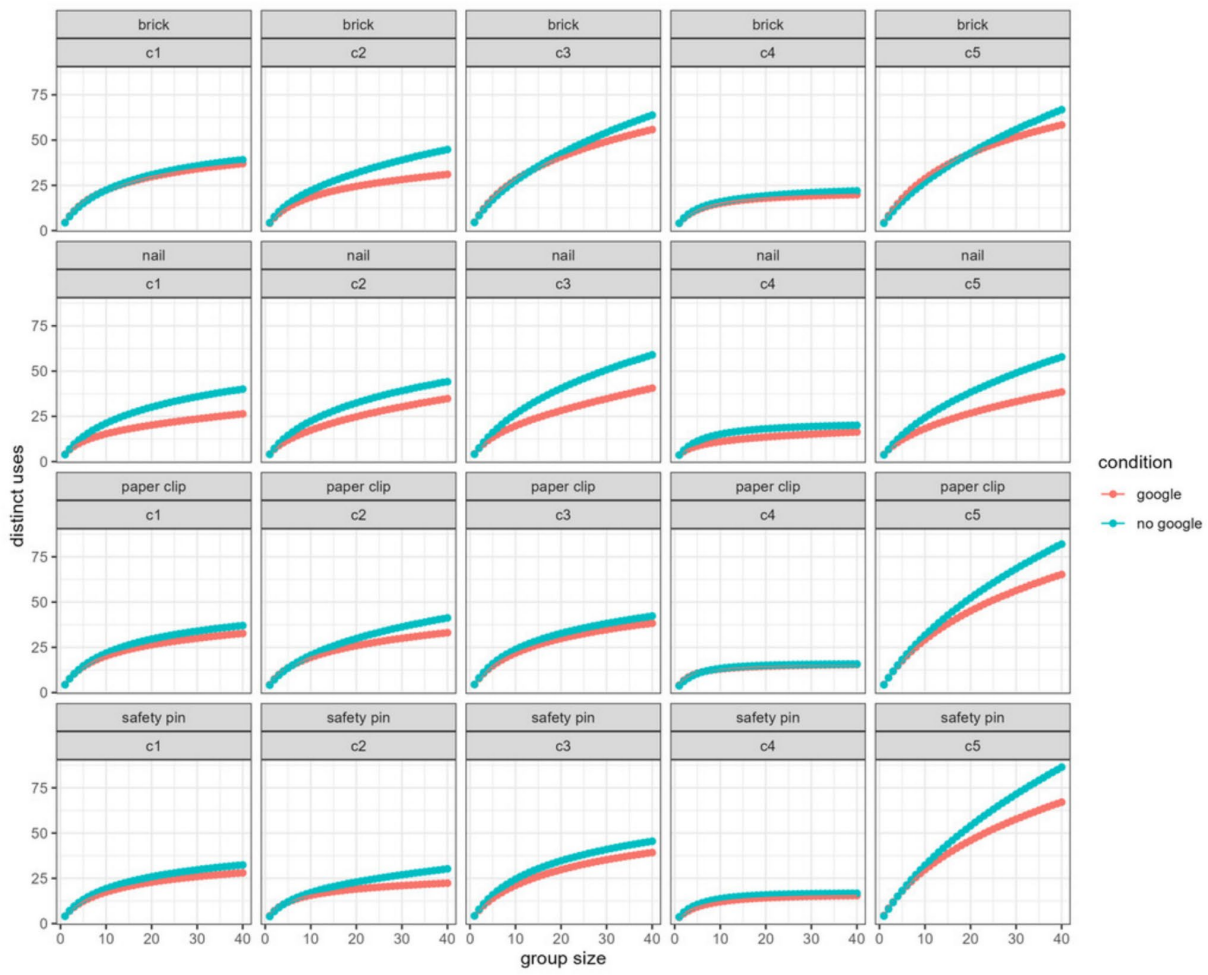
Oliva and Storm (2023) nominal group use generation results

As in Study 1, for the Google and non-Google groups separately, we created nominal groups by drawing 10,000 samples of groups of size *k* (without replacing individual participants within a sample), for groups of varying size (*k* ranging from 1 to 40). We then calculated the average number of distinct uses generated across all group members for each nominal group. Across five coders and four items, for a total of 20 analyses, we replicated the basic pattern of Study 1: As the group size increased, a creativity advantage for non-Google nominal groups emerged. While the magnitude of this advantage varied across coder and item, the general trend was observed in 20 out of 20 analyses. This suggests that the phenomenon is robust to differences in coding scheme and specific items being tested (see Fig. 4 for a visual representation of these results).

**Fig. 5:**
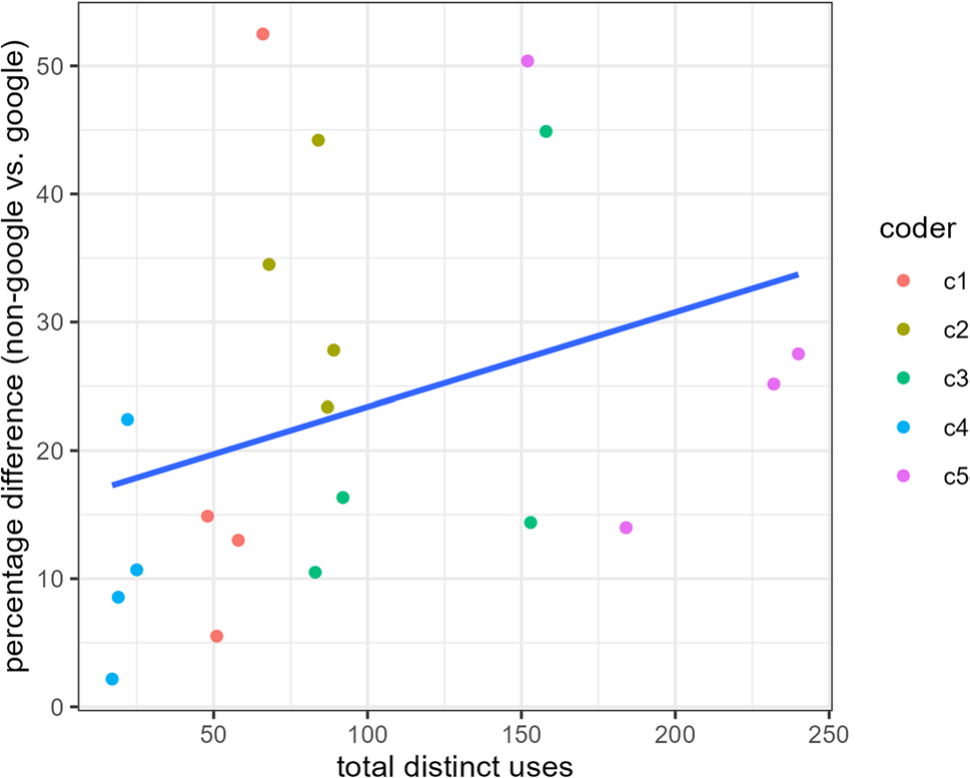
Non-Google percentage increase (relative to Google group) as a function of total distinct uses for *k* = 40. (Color figure online)

While the phenomenon emerged for all of the analyses, it is worth noting that the trends were stronger in some instances than others. In particular, coding schemes that yielded larger numbers of distinct categories tended to exacerbate the advantage of groups of non-Google users over groups of Google users. Figure 5 plots the percentage difference between the Google and non-Google groups as a function of how many categories a coder identified for a given condition for groups of *k* = 40. This is a post hoc, underpowered test (*n* = 20; 5 coders × 4 items), and the pattern does not reach conventional standards of statistical significance (β = 0.076, *p* = 0.13), so these results should be interpreted with extreme caution but nonetheless suggest that the more unique categories are observed, the more likely the unique category comes from a non-Google user, replicating the trends from Study 1.

In addition to the aforementioned analyses, we also explored how the use of the internet affects the likelihood of a group of individuals happening upon a truly unique or paradigm shifting idea.^6^ Of the thousands of answers in the Oliva and Storm (2023) data set, only 20 received perfect 5 out of 5 ratings from their judges on creativity—we define these as the *best* ideas. Ninety-five percent of those ideas came from participants without access to Google. Unsurprisingly, nominal groups without access to the internet were also far more likely to include the best ideas.

## Discussion

In this paper, we explored the relationship between creativity and internet use. Adopting an alternative uses paradigm, we found that when Google was not particularly useful (i.e., yielded few helpful results), or the number of uses that an individual could generate was capped by the experimental paradigm (i.e., users were only allowed to generate 5 ideas, cf. Oliva & Storm, 2023) there was no difference in creative fluency (number of items generated) between individual participants who used Google and those who do not. However, when participants were clustered into nominal groups, groups consisting of non-Google users outperformed Google users.

We also found that when Google was useful (i.e., yielded many helpful results) and there was no cap to the number of uses individuals could generate, then individuals who used Google outperformed those who did not on creative fluency. When participants were clustered into nominal groups, the advantage for using Google shrunk as the size of the group increased, until eventually larger nominal groups without Google outperformed nominal groups with Google. This appears to be due to the fact that Google users came up with the same common answers, often in the same order, as they relied on Google, while non-Google users came up with more distinct answers. The aggregation of these unique answers eventually overwhelms the advantage that Google provides individuals.

The pattern of larger nominal groups who did not have access to Google outperforming nominal groups who did have access to Google on creative fluency was quite robust. It was observed across six different items—including both items that are standard for the alternative uses task, and items that are nonstandard for the alternate uses task. It was observed across classification schemes generated by six independent coders—schemes which dramatically varied in terms of how narrowly the categories were defined (e.g., 21 vs. 157 categories for the same item). It was observed in two independent labs, each with variations in how the alternative uses task was instantiated. It was observed in all of the 22 test cases that we explored.

Similarly, when creativity was operationalized as effectiveness, for seven out of eight variations of our analysis, the same pattern emerged as for creative fluency (with the results inconclusive for the last cell). In contrast, the results were less robust for other operationalizations of fluency: novelty and subjective creativity. While in general, non-Google-using groups outperformed Google-using groups, the results were somewhat depended on the threshold used for what constitutes a high-quality answer, the coder, and the object of brainstorming. Thus, the effects of internet use on those operationalizations remains an open question, ripe for future exploration.

These results appear to be driven by the fact that when Google provided useful examples (e.g., uses for umbrellas), participants with access to Google conformed to those examples leading to homogeneity in the ideas generated. Surprisingly, when Google did not provide useful examples (e.g., uses for shields) participants with access to Google showed greater heterogeneity in ideas generated, relative to participants who did not have Google access (see Fig. 2). This unexpected pattern is difficult to explain—perhaps suggesting that participants who expect assistance but do not receive it are more motivated to think creatively. Future research should attempt to probe this anomalous finding more deeply.

### Cognitive implications

The patterns observed in the present data are similar to research on fixation effects (Kohn & Smith, 2011). Classic work in functional fixedness has shown that when a particular solution to a problem comes to mind, people often struggle to think of alternative approaches to the problem (e.g., Duncker, 1945; Maier, 1931). Similarly, when designers are provided with exemplars of a product with nontrivial flaws, they tend to include those flaws in their own designs (which does not happen in the control condition where no examples are shown; Jansson & Smith, 1991). When participants are primed with certain ways of thinking about problems, that tends to constrain their approach to generating ideas and solutions (Rietzschel et al., 2007). In creative generation tasks, participants who are primed with a subset of ideas will often generate just as many solutions as unprimed participants, but their answers show greater conformity (Kohn & Smith, 2011). Thus, as in the present paper, increasing nominal group size has greater effects when participants were not primed with examples before brainstorming. Interestingly, previous research has shown that explicitly instructing participants not to conform to provided examples does not reduce conformity (Smith et al., 1993), suggesting that these effects are not due to people viewing the examples as implicit recommendations of what good answers would look like. Instead, primes appear to be influencing how participants represent the problem space.

These fixation effects are related to part-set cuing phenomena in memory, in which people’s recall of exemplars of a category is inhibited by exposure to example category members (e.g., Marsh et al., 2004; Nickerson, 1984; Slamecka, 1968). Part-set cuing is often mechanistically explained as due to output interference, where cuing an example increases the strength of its memory trace, and thus outcompetes other category members during retrieval, effectively blocking the recall of other items (cf. Raaijmakers et al., 1981; Roediger & Neely, 1982; although other accounts emphasize inhibition of competing exemplars rather than strengthening the memory trace of primed examples; cf. Anderson et al., 2000).

To our knowledge, this is the first evidence of fixation effects being induced by internet search. As similar search terms will yield similar results, use of the internet for assistance in ideation can serve to focus people on the same small subset of the conceptual space. While that subspace is likely to mostly include good ideas (which is why they rose to the top of the search order), it could preclude people’s ability to generate particularly unique and novel ideas.

This overall pattern suggests that a tragedy of the commons might exist for creativity. While individuals can do better when using Google, the performance of the collective can be worse. Many of the large problems facing society do not have obvious solutions (or else they would have been solved already); such problems require outside-the-box thinking, something that we find is inhibited by internet use. Because most people frequently use Google, this suggests a barrier to successful resolution of society’s greatest challenges. Indeed, we found that 95% of the 20 “best” answers from the Oliva and Storm (2023) data set were generated by non-Google users, and thus groups that did not use Google were much more likely to generate the best answers. However, with only 20 observations of “best answers,” there is lot of uncertainty in our estimates of the proportion, and these results should be interpreted cautiously.

### Limitations and caveats

There are a number of caveats that should be taken into account when evaluating these studies. There is inherent arbitrariness in any classification scheme (e.g., is “use to decorate a room” distinct from “decoration in a photo shoot”?), and it is possible that the specifics of the coding scheme could have affected the results, even though the coders were blind to condition and hypothesis. Indeed, we found suggestive evidence that the observed effects were larger for coding schemes that had larger numbers of categories (see Fig. 4). Moreover, when exploring novelty and subjective wholistic evaluations of fluency, we found that the results varied somewhat by coder decisions. However, this concern is somewhat mitigated by our strategy of allowing different coders to use different, independent classification schemes, and the fact that the pattern of results for creative fluency and effectiveness, while larger for some coding schemes than others, was robust across every one of the coding schemes used.

The studies examined here were run on populations of undergraduates at highly selective research universities; there is no guarantee that the results would generalize across the population at large. However, the more diverse the population being sampled, the more likely that they will generate diverse creative responses, and this would be particularly true if they are not biased by the results generated by Google—suggesting that the results would only be stronger with a more diverse sample.

The two objects run in our primary study (shield and umbrella) were chosen because Google would be unhelpful and helpful, respectively, in generating alternative uses. However, those objects differ from one another on innumerable dimensions that confound interpretation of the results. For example, umbrellas are more common in everyday life, have more distinct parts, and form the title of a Rihanna song (Rihanna, 2007). The four additional objects from the reanalysis of the Oliva and Storm (2023) data have issues with ceiling effects, making it hard to draw strong inferences about how different types of objects might yield different results. Future studies should explore more objects to further elucidate these patterns of results.

In this study, we imposed a 3 min time constraint on participants to generate items. It could be that this time constraint influenced the pattern of results. The literature on time constraints and creativity is mixed. For example, while Liikkanen et al. (2009) found that time constraints actually increased participants’ ability to come up with novel solutions, Savage et al. (1998) found that participants had fewer ideas under time constraints. To our knowledge, no previous research has examined what happens to creativity when time constraints interact with internet use. Creativity could be reflected in how the internet is used, both in terms of how long participants use the internet and also what search terms they use, and in the strategies they use for perusing webpages. Especially with very short time constraints, participants might not have time to conduct an effective internet search, thus artificially creating the appearance that the internet harms creativity. While the 3-min time limit that we used in this study is unlikely to be short enough to preclude effective internet use, future research should explore what happens to creativity under varying time regimens.

There are many different digital tools that could conceivably affect creativity, and they may not all have the same cognitive consequences. In particular, large language models (LLMs) have the ability to generate large numbers of examples of various concepts, which could support creative thought. However, early studies on the use of LLMs have shown that writers using LLMs show less linguistic and semantic diversity than writers working without LLMs, particularly at the collective level (Moon, 2024). Similarly, consultants showed less creativity in consulting tasks when working with LLMs (Dell’Acqua, et al., 2023). Thus, the benefits of LLMs for creativity enhancement should be considered critically and empirically, and may benefit from scaffolding interventions such as the Delphi technique (Dalkey & Helmer, 1963).

## Conclusion

The way we think is influenced by the tools we use. Digital tools are being developed at an exponential rate with potentially great promise and great peril. In this paper, we showed that access to search engines and the internet can facilitate individual creativity while simultaneously impairing collective creativity. How that will affect society is still an open question, but the findings here suggest that you may not want to consult the internet when trying to brainstorm the answer.

## Data Availability

All study material and data are publicly available on OSF: https://osf.io/wsjqx/.

## Footnotes

1 Although the search for knee braces still yields mostly responses regarding joint protection: leg protection, compression garment for other joints, weightlifting accessory, fashion statement, proprioception training tool.

2 While we had initially intended to hire a second coder to establish interrater reliability, the fact that the finding was so robust across coders and categories for the reanalysis of Oliva and Storm (2023; see below) obviated the need.

3 Regressions are weighted by the number of uses, not the number of participants responding with the use. As an example, the 31 distinct “singleton” uses (only devised by a single participant) have a regression weight of 31. Regression weights match the size of the points in Fig. 2, and correspond with the “*n*” count.

4 Six coders were originally recruited; one coder completed categorization on only a single file and was thus discarded.

5 After an initial round of coding distinct responses, coders were asked to de-duplicate instances where the same verbatim response was given multiple distinct codes. Coders were also given latitude to subjectively decide whether a brainstormed response ought to be treated as a nonlegitimate use; such instances were treated as nonresponses at the coder level.

6 We thank the reviewers of a previous iteration of the manuscript for suggesting this insightful analysis.

## Code availability

All analysis scripts are publicly available on OSF: https://osf.io/wsjqx/.

## Declarations

### Artificial intelligence

This article was written using Google Docs and Microsoft Word, which both have AI generated wording suggestions (i.e., autocompleting words as they are being typed) and AI based spell-check and grammar-check. After the article had been written, GPT was used to check for typos, and to format the reference section. Portions of statistical analysis scripts were drafted using LLM; all AI-generated code was carefully checked for accuracy.
